# Rotating Magnetic Field-Assisted Reactor Enhances Mechanisms of Phage Adsorption on Bacterial Cell Surface

**DOI:** 10.3390/cimb44030088

**Published:** 2022-03-17

**Authors:** Bartłomiej Grygorcewicz, Rafał Rakoczy, Marta Roszak, Maciej Konopacki, Marian Kordas, Agnieszka Piegat, Natalia Serwin, Elżbieta Cecerska-Heryć, Miroslawa El Fray, Barbara Dołęgowska

**Affiliations:** 1 Department of Laboratory Medicine, Chair of Microbiology, Immunology and Laboratory Medicine, Pomeranian Medical University in Szczecin, Powstańców Wielkopolskich 72, 70-111 Szczecin, Poland; martaroszak95@gmail.com (M.R.); maciej.konopacki@zut.edu.pl (M.K.); natalia.serwin@pum.edu.pl (N.S.); elzbieta.cecerska.heryc@pum.edu.pl (E.C.-H.); barbara.dolegowska@pum.edu.pl (B.D.); 2 Department of Chemical and Process Engineering, Faculty of Chemical Technology and Engineering, West Pomeranian University of Technology, Szczecin, Piastów Avenue 42, 71-065 Szczecin, Poland; rafal.rakoczy@zut.edu.pl (R.R.); marian.kordas@zut.edu.pl (M.K.); 3 Department of Polymer and Biomaterials Science, Faculty of Chemical Technology and Engineering, West Pomeranian University of Technology, Szczecin, Piastów Avenue 42, 71-065 Szczecin, Poland; agnieszka.piegat@zut.edu.pl (A.P.); mirfray@zut.edu.pl (M.E.F.)

**Keywords:** adsorption kinetic, bacteriophage, bacteriophage production, bioreactor processes, rotating magnetic field

## Abstract

Growing interest in bacteriophage research and use, especially as an alternative treatment option for multidrug-resistant bacterial infection, requires rapid development of production methods and strengthening of bacteriophage activities. Bacteriophage adsorption to host cells initiates the process of infection. The rotating magnetic field (RMF) is a promising biotechnological method for process intensification, especially for the intensification of micromixing and mass transfer. This study evaluates the use of RMF to enhance the infection process by influencing bacteriophage adsorption rate. The RMF exposition decreased the t_50_ and t_75_ of bacteriophages T4 on *Escherichia coli* cells and vb_SauM_A phages on *Staphylococcus aureus* cells. The T4 phage adsorption rate increased from 3.13 × 10^−9^ mL × min^−1^ to 1.64 × 10^−8^ mL × min^−1^. The adsorption rate of vb_SauM_A phages exposed to RMF increased from 4.94 × 10^−9^ mL × min^−1^ to 7.34 × 10^−9^ mL × min^−1^. Additionally, the phage T4 zeta potential changed under RMF from −11.1 ± 0.49 mV to −7.66 ± 0.29 for unexposed and RMF-exposed bacteriophages, respectively.

## 1. Introduction

The electromagnetic field (EMF) has been widely used in different areas of technical applications or bioprocessing [1]. The utilization of different kinds of EMFs to process intensification was excessively studied in the past. However, studies on microorganisms exposed to force fields mainly focused on the static magnetic field (SMF) generated by permanent magnets or alternate magnetic field (AMF) generated by coils of different construction. However, there are many other field types that should be investigated [2,3,4]. In contrast to the SMF, the AMF changes in time with a specific frequency, inducing vulnerable particles and ions movement. Thus the AMF impact on microorganism’s metabolism and living matter processes is very different [5,6]. The AMF may also be an alternative to mechanical mixing [2,7] to increase mass and heat transfer, especially when high shear stress created by a mechanical agitator can be very harmful to live cells. The particular case of AMF is the rotating magnetic field (RMF) generated by the superposition of three shifted AMFs that results in a single circulating force field around its axis. It has been proved that the application of RMF may enhance mass transfer processes [8,9,10]. Moreover, previous works showed that RMF might impact the microorganism’s growth dynamics and alter the metabolic activity that can enhance biomass and bio-compounds production depending on the type of utilized cells and operating conditions [11,12]. RMF exposition showed an increase in the *Escherichia coli* and *Staphylococcus aureus’s* viability, triggering Gram-positive and Gram-negative bacteria growth, modification of bacterial cellulose parameters, and changed biofilm antibiotic susceptibility [12,13,14].

Bacteriophages are one of the most widespread groups of organisms worldwide [15,16]. Those bacterial cells infecting viruses are characterized by a high specificity [17,18]. One of the most promising uses of bacteriophages in treating multidrug-resistant bacteria infection is when antibiotics fail [19]. Additionally, considering the rapidly increasing bacteriophage application in food production and processing, diagnostics, nanomaterial production, animal husbandry, and veterinary medicine [20,21,22,23], it is clear that the near future will need improvement of bacteriophage production processes and therapeutic effectiveness [24,25].

The present work’s main goal was to analyze the RMF influence in the magnetically assisted bioreactor on the bacteriophage adsorption kinetics to understand this phenomenon better and perform further optimization procedures.

## 2. Materials and Methods

### 2.1. Rotating Magnetic Field Assisted Reactor (RMF-AR)

**Figure 1:**
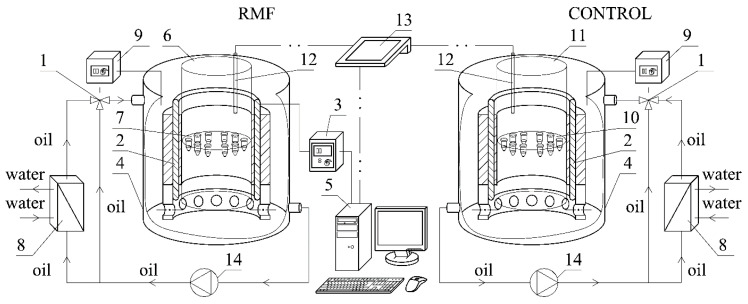
Experimental set-up: 1—cooling system; 2—RMF generator; 3—ac transistorized inverter; 4—vessel; 5—personal computer; 6—glass container; 7—probe; 8—heat exchanger; 9—thermostat; 10—control probe; 11—control container; 12—temperature sensor; 13—multifunctional computer meters; 14—circulation pump.

The experimental setup used in this study is presented in Figure 1. This apparatus consists of a cooling system (1) and an RMF generator (2). In the current study, the RMF was generated by a three-phase stator equipped with the set of coils (windings) collected from a squirrel cage type of an induction motor. The stator consists of three coils sets powered by each electric phase and situated around a single axis every 120°. The current flow through the coils creates the electromagnetic fields that interfere with other, and then by fields superposition, the RMF occurs. The created RMF has a single resultant vector of force in which direction changes with time (rotating around a center axis), but the field’s intensity is constant. Nevertheless, the field intensity changes with distance: it decreases from the coils to the center of the generator in the horizontal direction. Moreover, intensity changes are observed concerning the distance from the middle of height in the vertical direction. The RMF generator (2) was connected with an AC transistorized inverter (3). The used inverter allows to set and change AC power frequency and voltage. The output frequency (the voltage frequency between the output terminals of the inverter) and output voltage (the voltage between the output terminals of the inverter) may be treated as adjustable parameters. The RMF generated in the system may be controlled through power supply adjustment because changes of the electric voltage affect the value of field frequency proportionally. The inverter was connected with a computer (5) with the proper software to specify and control all current parameters and the RMF frequency. In the case of the present studies, the current output frequency was 50 Hz (max. magnetic field induction was *B_max_* = 45 mT).

The temperature of the whole system (especially culture medium) was controlled by a proper cooling system (1), equipped with Pt-100 type temperature probes. Because the RMF generator produces heat through powered coils, a thermostat (9) was utilized to control temperature increase. For typical cases, the oil from the generator tank was pumped in a loop from the bottom to the top of the tank; however, when the temperature was raised, oil was transferred through the plate heat exchanger supplied with cold tap water to lower its temperature. The cooling system’s proper adjustment and the controller allowed to obtain stabilized thermal conditions at 37 ± 0.2 °C. Additionally, the temperature fluctuations inside the glass (6) and control (11) containers were measured by using the temperature sensors (12) connected with the multifunctional computer meter (13).

### 2.2. Bacteria and Bacteriophage Used in Study and Culture Condition

This study used *Escherichia coli* C600 and *Staphylococcus aureus* ATCC6538 as target bacteria cells. Bacterial strains were cultured in Luria–Bertani broth (LB, 10 g of NaCl, 10 g of tryptone, 5 g of yeast extract, 1000 mL of water; pH 7.4) with shaking (160 rpm), at 37 °C, overnight. For the bacteria enumeration, serial dilutions in the phosphate-buffered saline buffer (PBS, 137 mM NaCl, 2.7 mM KCl, 10 mM Na_2_HPO_4_, 1.8 mM KH_2_PO_4_; pH 7.4) were prepared and spread on LB supplemented with 1.5% agar and incubated at 37 °C overnight for the colony counting. The number of cells was expressed in CFU × mL^−1^.

For bacteriophage T4, and bacteriophage vB_SauM_A (bacteriophage described by Łubowska et al. [26]) quantification, phage stocks were serially diluted in SM buffer (100 mM NaCl, 8 mM MgSO_4_, 50 mM Tris-Cl, 0.01% (*w*/*v*) of gelatine; pH 7.5, all chemicals were purchased from Sigma-Aldrich Co., LLC, MO, USA). Afterward, the 100 μL of phage suspension, 100 μL of the bacterial culture (OD_600nm_: 0.5), and 3 mL of LB (0.7% of agar) were mixed and poured onto LB agar plates. The plates were incubated overnight at 37 °C. The number of plaques was performed for the appropriate dilutions. The count between 30 and 300 plaques was considered valid. Phage counts were expressed as PFU × mL^−1^.

### 2.3. Adsorption Rate Protocol under RMF Condition

Phage adsorption experiments were conducted according to Kropinski [27] and Lee et al. [28] with minor modifications. Bacterial strains were grown to OD_600nm_ = 0.5 (with or without RMF exposition) in LB medium. Next, bacterial culture was mixed with a suspension of bacteriophage at the multiplicity of infection (MOI) equal to 0.01. The mixture was incubated at 37 °C. Samples were collected in 2-min intervals, mixed with chloroform (approx. 10%), and centrifuged at 15.7× *g* for 1 min. The decimal dilution of unadsorbed-phage-containing supernatant was prepared for the phage titration determined in plaque assay as described above. The adsorption rate was calculated according to Kropinski [27].

(1) $$k=\frac{2.3}{B\;t}\log_{10}\left(\frac{P_{0}}{P}\right)$$

where:

*B*—bacterial initial count, [CFU × mL^−1^];
*k—*adsorption rate constants;
*P*_0_*—*phage initial counts, [PFU × mL^−1^];
*P—*phage count at time point, [PFU × mL^−1^];
*t—*time, [min].

### 2.4. Outer Membrane Charge Assessment

Cytochrome C binding assay was used to assess the outer membrane charge by measuring the amount of cationic Cytochrome C binding to cells, as previously described by Beebout et al. [29]. Planktonic cells were extracted from mid-logarithmic phase cultures. Cells were normalized to OD_600_ = 2.0 and washed twice in 20 mM MOPS (pH 7.0). Cationic equine Cytochrome C (Merc, Darmstadt, Germany) was added to 0.5 mg × mL^−1^. Cells were incubated with Cytochrome C for 5, 10, and 20 min at 37 °C under exposure to RMF and in the control reactor. After incubation, cells were pelleted by centrifugation and unbound Cytochrome C was measured from the supernatant by quantifying absorption at 530 nm.

### 2.5. Zeta Potential and Diffusion Coefficient Measurement

The Zeta potential of *E. coli* cells and bacteriophage T4 treated with RMF was measured on a Zetasizer Nano ZEN 3600 (Malvern Instruments, Malvern, UK) at 25 °C. Moreover, the same device allowed us to perform the dynamic light scattering procedure to obtain microorganisms diffusion coefficient values. Obtained data are an average of six biological replicates with six measurements each.

### 2.6. Statistical Analysis

For all obtained experimental data statistical analysis, the two-way ANOVA was performed. *p* values of <0.05 were considered significant. All mathematical procedures were conducted using Statistica software (TIBCO Software Inc., Palo-Alto, CA, USA).

## 3. Results

### 3.1. Effect of RMF-AR on the Bacteriophage Adsorption

**Figure 2:**
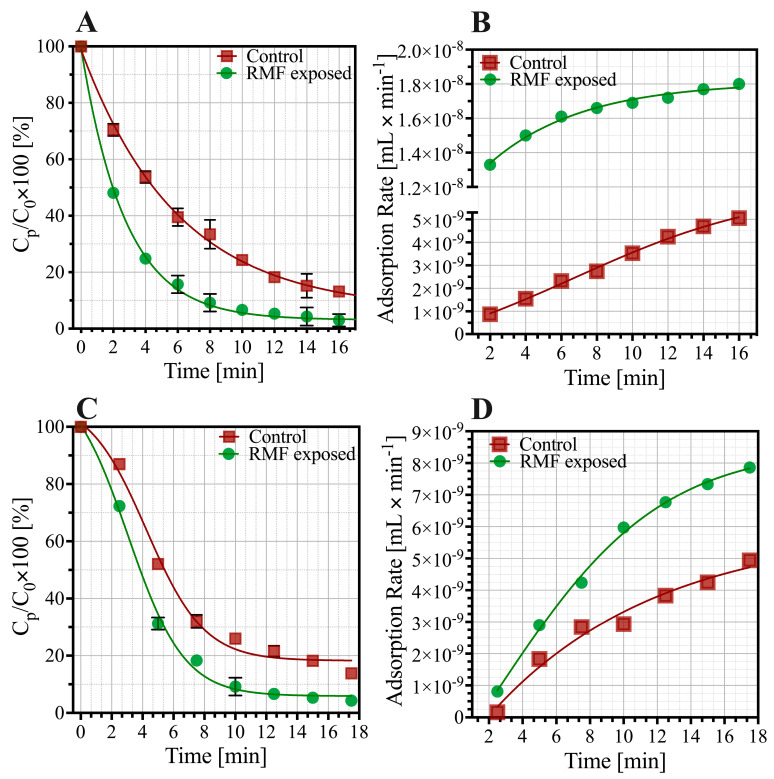
Influence of the RMF on bacteriophage adsorption process: (**A**) the percentage of unabsorbed T4 bacteriophages under control (red) and RMF exposed condition (green); (**B**) adsorption rate curves of RMF-exposed and unexposed bacteriophage T4; (**C**) the percentage of unabsorbed vB_SauM_A bacteriophages under control and RMF exposed condition (**D**) adsorption rate curves of RMF-exposed and unexposed vB_SauM_A. C_p_—number of phages on timepoint, C_0_-number.

The T4 bacteriophage’s adsorption on exponentially growing *E. coli* cells under RMF (B = 30 mT, f = 50 Hz) exposition was higher than adsorption under normal conditions. The obtained results showed that after 2 min incubation, approximately 30% of free virion was attached to the cells in the control sample. Adsorption effectiveness increased when RMF exposition was applied and resulted in attachment of approx. 52% of virions. After 4-min incubation, approx. 47% and 75% of free virions attached to bacterial cells in control and RMF-exposed groups, respectively (Figure 2A). In the case of Staphylococcus infecting phage, the percentage of adsorbed virion also changed. After 2 min incubation, 13% of phages adsorbed in control conditions. After applying the RMF exposition, after 2 min, 27% of free virions were adsorbed to host cells.

Phage T4 that adsorbed under RMF exposition showed significant decreases in the *t*_50_ (50% virion adsorption) and *t*_75_ (*p* < 0.05). Bacteriophage T4 *t*_50_ value decreased from 5.1 min to 2.2 min, and the *t*_75_ value from approx. 10 min to 4 min. Phage vb_SauM_A exposed to RMF also showed significant decreases in the *t*_50_ (decreased from 5.2 to 3.8 min) and *t*_75_ (reduced from approx. 9.2 to 6 min). Changes in the adsorption time influenced the adsorption rate. The detailed bacteriophages adsorption rates for each time point are presented in Figure 2B,D. Average adsorption rate increased in the case of RMF exposed bacteriophage T4 from 3.13 × 10^−9^ mL × min^−1^ to 1.64 × 10^−8^ mL × min^−1^. The adsorption rate of vB_SauM_A exposed to RMF increased from 4.94 × 10^−9^ mL × min^−1^ to 7.34 × 10^−9^ mL × min^−1^. The control sample showed that RMF exposition does not affect bacteriophage survival in the culture medium.

### 3.2. Effect of RMF-AR on Cytochrome C Binding on the Bacterial Cell Surface

According to adsorption changes, one possible explanation for the alterations in bacteriophage adsorption changes is that RMF-treated bacterial cells have changed the charge. To test this possibility, we measured the interaction of equine Cytochrome C with the cell exposed to RMF and in control reactors. Quantifying the binding of polycationic Cytochrome C that interacts electrostatically with the negatively charged bacterial cells can determine the relative cell charge [29]. In RMF-treated bacterial cells, we observe more efficient binding of Cytochrome C both in the case of *S. aureus* and *E. coli* cells. Thus, suggesting that RMF-exposed cells have a more negatively charged outer membrane under this condition than cells in the control reactor.

### 3.3. Effects of RMF-AR on the Bacteria and Bacteriophage Zeta Potential

The zeta potential measurements were carried out to investigate the electrostatic charges on the surface of bacteria and phages after exposition to RMF. The exposure of *E. coli* to RMF does not significantly change the zeta potential of cell surface charge. Under these conditions, unexposed *E. coli* cells were characterized by the average zeta potential of about −11.74 ± 0.62 mV, and RMF-exposed cells were found to have an average of about −11.13 ± 0.77 mV. Interesting findings are the effect of RMF on bacteriophage T4 zeta potential. The zeta potential values −1.1 ± 0.49 mV and −7.66 ± 0.29 for unexposed and RMF-exposed bacteriophages, respectively, showed a more significant positive charge of RMF-exposed than the unexposed bacteriophages.

The diffusion coefficient of RMF-exposed and non-treated *E. coli* was 0.13 ± 0.005 m^2^·s^−1^ and 0.18 ± 0.06 m^2^·s^−1^ with hydrodynamic radius 3.34 ± 0.57 µm and 3.64 ± 0.24 µm, respectively. However, this coefficient changed in the case of bacteriophage where values of 3.33 ± 0.15 m^2^·s^−1^ and 4.11 ± 0.12 m^2^·s^−1^ and the hydrodynamic radius were 145.62 ± 8.61 nm and 109.02 ± 2.86 nm for unexposed and RMF-treated bacteriophage T4, respectively, were found. An interesting result is the change of the bacteriophage zeta potential after RMF exposition. The bacteriophage virion’s electrophoretic studies suggest that the head is responsible for the phage virion’s negative charge, and the tail fibers are probably positively charged [30]. Obtained results showed that the zeta potential values of the virions changed from approx. −11.1 ± 0.49 mV to −7.66 ± 0.29 for RMF-exposed bacteriophages. Zemb et al. [31] showed that NaCl addition to freshwater increases the adsorption of bacteriophage T2 virions onto *Escherichia coli*.

**Figure 3:**
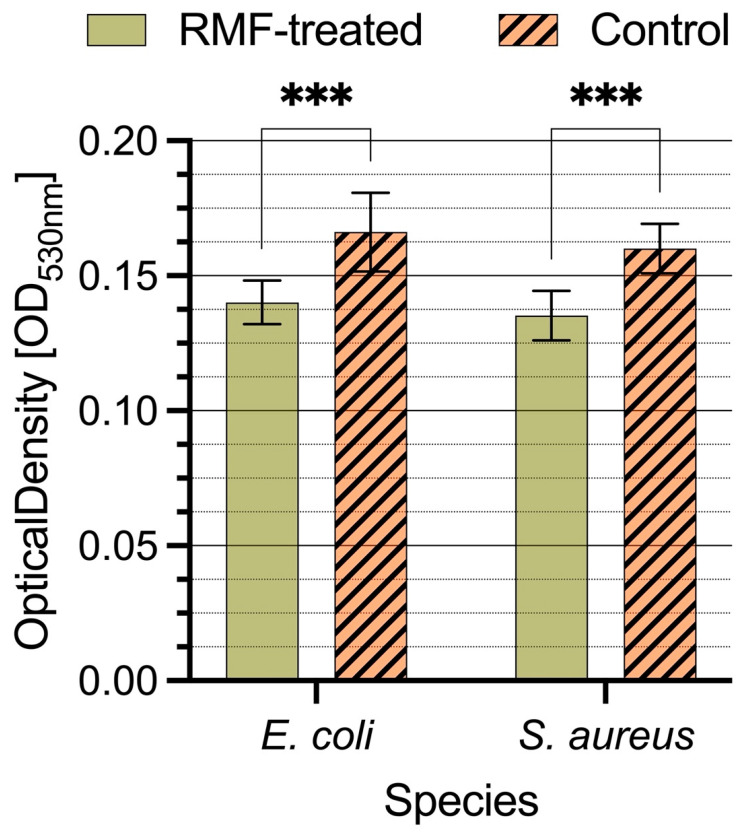
Influence of the RMF exposition on Cytochrome C binding to the bacterial cell surface. *** indicate statistically significant values.

Additionally, they showed that increased ionic strength of medium increases the zeta potential of bacteriophage T2. The mechanisms of RMF action are not fully elucidated, and we can interpret our results with caution. We hypothesize that increased mobility of positively charged ions affects the bacteriophage zeta potential by influencing the virion surface. Additionally, it should be noted that these ions, especially divalent ions, play an essential role in the bacteriophage infection process [32,33]. The increased mobility of these ions in the effect of eddy currents and micro-mixing may influence bacteriophage adsorption and proliferation, and the verification of this requires further research. The visualization of the concept of how the RMF affects the bacteriophage adsorption process is presented in Figure 3.

## 4. Discussion

The phage adsorption process on the host cell is interpreted as the attachment of the bacteriophage virions onto a surface of the cell. This process could be described as combining highly specific interactions between phages and bacterial cells. The kinetics of the diffusion process can influence the adsorption phenomenon, amount of electrostatic charges, and moves on the surface, especially Brownian-types. Still, it also can be altered by different bacteria morphology and physiology, bacteriophage mode of action, and biochemical interaction [31,34,35,36]. Delbruck (1940) describes the decrease in bacteriophage adsorption for bacteria in which motility was affected by environmental factors [37]. Observation showed that motile bacteria generated Brownian-type motion near their cells [38]. The generation of the Brownian movement could enhance bacteriophage adsorption through the energetic barrier [31]. According to this information, the first factor that explains phage adsorption process enhancement by RMF could initially be the RMF-mediated micro-mixing phenomena. The dissolved ions included in microbiological media, under RMF exposition, can cause the medium’s movement. Experimental data showed that RMF exposition enhances the liquid mixing processes [39]. Thus, the observed phenomenon might be explained by the micro-level dynamo concept [2,12]. Inside the electromagnetic field (e.g., RMF), any charged particles, such as ions, are forced to move by electromagnetic force, creating electric potential and generating local eddy currents, especially in water solutions such as culture medium [40,41]. The induced eddy currents may act like small permanent magnets and create local MFs around the charged particles. The additional influence of the electromagnetic field may cause the movement of such particles around their axis. Hristov (2010) called this phenomenon “micro-dynamos” that cause the micro-mixing effect. Thus, the application of the RMF may generate the circulation of charged particles inside the culture medium around the generator axis and the local micro-mixing effect.

For this reason, the RMF assisted apparatus can be treated as the electromagnetic mixer [42]. This kind of mixing may be an essential alternative to traditional mechanical agitators in microorganism’s cultivation thanks to its low shear stress. Moreover, previously conducted studies suggested that RMF exposition could alter the yeast and bacteria proliferation and metabolic activity [11,12]. It was found that RMF constant exposition (frequency 50 Hz, maximal induction 18 mT for 72 h) on yeast cells may improve the bioethanol production from sugar by increased yield and efficacy. The addition of magnetically vulnerable particles (Fe_3_O_4_) in the culture medium as a sample coating increased the mixing intensity, thus also improving the studied process [43].

**Figure 4:**
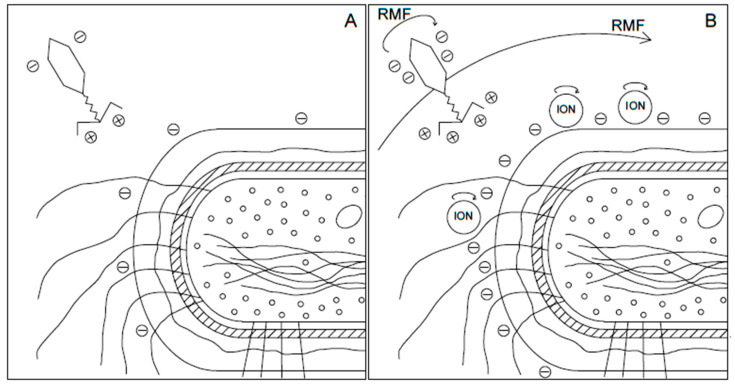
The visualization of the concept of RMF action on bacteriophage adsorption: (**A**) Adsorption process without RMF, (**B**) adsorption process with RMF.

Furthermore, previously obtained results showed that bacteria affected by the RMF exposition could be stimulated or inhibited, depending on the bacteria morphology, and so utilized intercellular transport mechanisms. In some instances, bacteria’s metabolic activity and growth rate were significantly increased. One of the possibilities is the facilitation of particle penetration through the bacteria membrane by RMF [12]. This mechanism can also improve the bacteriophage adsorption process by changing the cell surface charge on the bacteria cell surface. Additionally, bacteria with overregulated metabolism can metabolize faster and have a larger cell size, which can also impact bacteriophage adsorption [44,45]. The visualization of the concept of RMF action on bacteriophage adsorption id presented on Figure 4.

## 5. Conclusions

Present studies indicated that the adsorption process of T4 bacteriophage on the bacteria host’s surface could be improved by RMF exposition. It has been proved previously that the RMF system can act as an active micro-mixer in water solution systems such as bioliquids. The culture medium contains many magnetically vulnerable particles (e.g., ions), which are forced to move under the action of the external magnetic field, thus also creating a movement of liquid particles causing the micro-mixing phenomenon. It is well known that the mixing process can affect the adsorption process, which was observed during these studies. Moreover, the electromagnetic field may charge surfaces and an induced electric field. Due to the increased charges on the host cell surface and bacteriophage tail (see Figure 3), the adsorption of bacteriophage may be more accessible thanks to the stronger electrostatic forces and RMF-forced movement of the phage near the surface of the host cell, compared to the conditions without the RMF. Forthcoming works should concentrate on the influence of RMF on bacteriophages lytic activity and therapeutic effectiveness.
